# Severe Plastic Deformation of Fe-22Al-5Cr Alloy by Cross-Channel Extrusion with Back Pressure

**DOI:** 10.3390/ma11112214

**Published:** 2018-11-08

**Authors:** Radosław Łyszkowski, Wojciech Polkowski, Tomasz Czujko

**Affiliations:** 1 Faculty of Advanced Technology and Chemistry, Military University of Technology, 2 Urbanowicza, 00-908 Warsaw, Poland; radoslaw.lyszkowski@wat.edu.pl; 2 Foundry Research Institute, 73 Zakopiańska, 30-418 Cracow, Poland; wojciech.polkowski@iod.krakow.pl

**Keywords:** severe plastic deformation (SPD), cross-channel extrusion (CCE), back pressure (BP), numerical simulation (FEM), physical modeling technique (PMT)

## Abstract

A new concept of the cross-channel extrusion (CCE) process under back pressure (BP) was proposed and tested experimentally. The obtained by finite element method (FEM) results showed that a triaxial compression occurred in the central zone, whereas the material was deformed by shearing in the outer zone. This led to the presence of a relatively uniformly deformed outer zone at 1 per pass and a strong deformation of the paraxial zone (3–5/pass). An increase in the BP did not substantially affect the accumulated strain but made it more uniform. The FEM results were verified using the physical modeling technique (PMT) by the extrusion of clay billet. The formation of the plane of the strongly flattened, and elongated grains were observed in the extrusion directions. With the increase in the number of passes, the shape of the resulting patterns expanded, indicating an increase in the deformation homogeneity. Finally, these investigations were verified experimentally for Fe-22Al-5Cr (at. %) alloy using of the purposely designed tooling. The effect of the CCE process is the fragmentation of the original material structure by dividing the primary grains. The complexity of the stress state leads to the rapid growth of microshear bands (MSB), grain defragmentation and the nucleation of new dynamically recrystallized grains about 200–400 nm size.

## 1. Introduction

Plastic working is one of the most popular and cost-effective methods to improve the mechanical properties of structural materials. A structural transformation occurs upon the plastic working processing, leading to a homogenization and a grain structure refinement and, thus, an increase in the mechanical strength. However, the level of strain imposed to the material is limited by its cohesion strength and is especially important in the case of materials with a lowered deformability.

In conventional processing techniques, e.g., rolling or compression, the maximum strain value is limited by the thickness/quantity of material in the deformation axis. However, severe plastic deformation (SPD) methods allow a much higher accumulated strain, due to the introduction of complex deformation schemes [1,2]. The SPD techniques are based on the domination of compression strain and a cyclically changed strain path and are actually considered the most efficient method of fabricating ultrafine grained metals and alloys [3,4,5]. The SPD methods are characterized by the imposition of strain values that significantly exceed those introduced in conventional processing. However, a successful implementation of the SPD techniques requires the proper selection of both the processing conditions and tool design [6,7].

**Figure 1:**
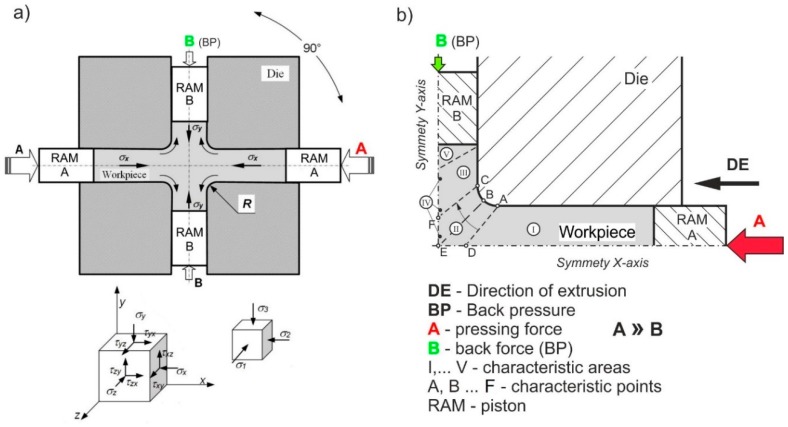
Scheme of (**a**) cross-channel extrusion with back pressure and (**b**) adopted analysis system.

Cross-channel extrusion (CCE) is a relatively seldom used method of deformation that belongs to the group of SPD processes. Unlike methods, such as high pressure torsion (HPT), where very large deformation values can be obtained in a small sample volume [8], extrusion-based methods allow one to obtain bulk materials of a certain size to ensure their practical use in the technique [9,10]. The principles of CCE processing (Figure 1) are based on the materials extrusion (by using a punch A that moves along the *X*-axis) with a perpendicular direction of the material flow (along the *y*-axis). This method allows a high accumulated strain to be obtained upon a 90° rotation of the die [11,12] without removing the material between subsequent deformation passes. Due to the continuous nature of the process and a possibility of automation, this method might be potentially adopted to industrial conditions [9,13].

Nevertheless, despite the presence of a triaxial stress state, the application of the CCE method to hardly deformable materials, such as Fe_3_Al-based intermetallic alloys, is still limited due to these materials’ lowered ductility [14,15,16,17,18,19,20]. Therefore, the main goal of the CCE process is to increase the level and extent of imposed hydrostatic strain by inhibiting a material’s flow and, thereby, to produce a uniform shear deformation that prevents defect formation in the workpiece [21,22,23,24]. This assumption may be successfully accomplished by implementing a second punch (Figure 1b) that gives a back pressure (BP) and allows a controlled limitation of the material’s lateral flow. To date, only a few cases of the BP effect have been reported [25,26,27,28,29], and its role has not been fully clarified.

Both the cross-channel extrusion and equal channel angular pressing (ECAP) methods belong to side extrusion-type processing. The former is described as a double axis technique, and the latter is assigned to side extrusion methods [6]. In these processes, pure shear deformation can be repeatedly imposed to a material such that an intense plastic strain is produced without any change in the cross-sectional dimensions of the workpiece. The die design in CCE methods is quite similar to that used in the ECAP-A—it may be considered a combination of four ECAP channels connected by their internal surfaces (as ╬). However, the main difference is that in the CCE process, a material is introduced to the die (along the *X*-axis) and leaves it in two opposite directions (along the *y*-axis) [9,10], but there is only one flow direction in the ECAP method. Consequently, a problem with filling an outer corner of the die (around a point E in Figure 1b), as is commonly observed in the ECAP method [22,25,27,28], is strongly limited in CCE processing. Moreover, this adverse effect may by additionally inhibited by using BP. Moreover, in order to alleviate deformation conditions and thereby limit the possibility of defects in hardly deformable materials [21,22], the sharp internal corner has been replaced by ABC arc.

In both methods, a shear strain is involved in a deformation mode. However, in the CCE method, the friction between a sample and die’s walls, as well as the load is smaller, because a lower die’s wall is replaced by a processed material that plays the same role as a movable die wall in the output channel of classical ECAP [13,28,30]. Thus, in addition to the imposed shear strain, a high hydrostatic compression occurs, which leads to a higher accumulated strain and prevents a material’s cracking.

Despite the obvious advantages, the successful implementation of numerical methods requires an accurate knowledge of the material constitutive equations, process mechanics and frictional conditions. Inaccurate information regarding any of these parameters may lead to highly erroneous results. Therefore, this method requires validation, preferably based on real processes.

The physical modeling technique (PMT) is an alternative analysis method that can provide information on the plastic flow of metals, load predictions and strain distributions in metal forming processes. Using a suitable material, we can clearly observe the material flow pattern during processing, the effect of mold wall friction and a true-to-nature representation of the starting microstructure of the feedstock, all of which are possible when using this method. Usually, for modeling, materials based on the plastic mass (wax, modeling clay) [31,32,33], or ductile metals (Pb, Zn, Cu and other), are used [34,35,36]. Regardless to clearly visible differences between materials applied to PMT technique and regular constructive materials, in particular much higher plasticity, the mentioned above materials fulfill the condition of similarity and proportionality [37]. Due to this fact the PMT method allows for the qualitative and quantitative evaluation of the modeling process.

A new solution to the problem of low-ductility materials processing by CCE die has been proposed recently. Introduction of back pressure has a significant impact on the ability of the process, what was confirmed by finite element method (FEM) simulations and physical modeling of this process. The current paper focuses on checking the engineering feasibility of this idea by carrying out a laboratory experiment and investigating of macrostructure in terms of the possibility of defects occurrence.

## 2. Experiment Details

The results of our previous study [15,16] on the compression of Fe-22Al-5Cr intermetallic alloys carried out with GLEEBLE 3800 plastometric testing device were used to build a mathematical description of the model by FormFEM software (ITA Ltd., Ostrava, Czech republic). A rigid-plastic body model described by the following equation was used:

(1) $$\int_{V}\bar{\sigma }\;\delta \dot{\bar{\varepsilon }}\;dV+\int_{V}\sigma_{m}\;\dot{\delta \varepsilon_{\nu \;}}dV+\int_{V}\delta \sigma_{m}\dot{\;\varepsilon_{\nu }}dV-\int_{S_{F}}F_{i}\;\delta u_{i}\;dS=0,$$

where:
$\bar{\sigma }$—stress intensity*V*—volume$\sigma_{m}$—average hydrostatic stress*S*—surface area$\dot{\varepsilon }$*_ν_*—strain rate of material’s volume*F_i_*—heat flow$\dot{\bar{\;\varepsilon \;}}$—strain rate intensityδ—material constant.

An incompressibility condition was fulfilled by the assumption of Lagrange multipliers method. A heat quantity generated upon the deformation process was calculated based on Fourier equation, and then correlated with a mechanical behavior by the following equation:

(2) $$\int \bar{\sigma \;}\dot{\bar{\varepsilon }\;}dV=q.$$

where:

*q*—an amount of heat generated in the body deformed as a result of the deformation work.

Then, rheological parameters of the Hansel-Spittel equation for the strain rate of 0.01 s^−1^ and the assumed temperature range were taken from the conducted compression tests as follows:

(3) $$\sigma =1115.06e^{-0.002294T}\cdot \varepsilon^{0.120494}\cdot 0.01^{0.01026}.$$

where:

σ, ε—stress, strain.

Two-dimensional FEM simulations were performed to investigate the effect of BP on the deformation behavior of Fe-22Al-5Cr (at. %) intermetallic alloy during the CCE process. The assumed simulation conditions included various values of pressure and numbers of passes upon processing. The FEM models of a die and punch were developed and then limited to a representative quarter. The cross-section of the die channels was 10 mm square. The model assumed a continuity of material along the horizontal or vertical symmetry axis by attaching moving supports to all axial nodes. A movement range of the punch, heat transfer and surface conditions (e.g., coefficient of friction) between contacting parts were determined [38]. The BP was modeled by introducing a proper value of the pressure on a face plane of a sample. As a consequence, this plane was not flat, as in a real case, but possessed a lens-shaped free surface. The BP values were set as 100 MPa and 500 MPa, which corresponded to 10% and 50% of the main pressing pressure, respectively. The simulation was carried out for CCE process at room temperature.

The physical modeling technique PMT can be carried out in two ways. The first method involves the formation of an ordered structure with a characteristic pattern and then analyzing the deformation. For this purpose, a sample with alternating plates or cubes in different contrasting colors was prepared [32,39,40,41,42,43]. The second method, presented in this paper, is the formation of a sample of randomly distributed particles and observing the arrangement and creation of characteristic patterns [31,37]. Observing the development of the grain structure of deformed material is also possible in this much easier preparation technology.

For this purpose, extruded homogenized clay in five contrasting color rods of Ø1 mm was used. The cut rods with 1 mm long grains were mixed, pressed into a cylinder sample size of Ø8 × 36 mm. After being placed in a channel die, the sample was extruded at a rate of 5 mm/min and then thermally fixed. To evaluate the process of deformation performed by conventional methods on the longitudinal and cross section of the samples, they were subjected to macroscopic analysis.

**Figure 2:**
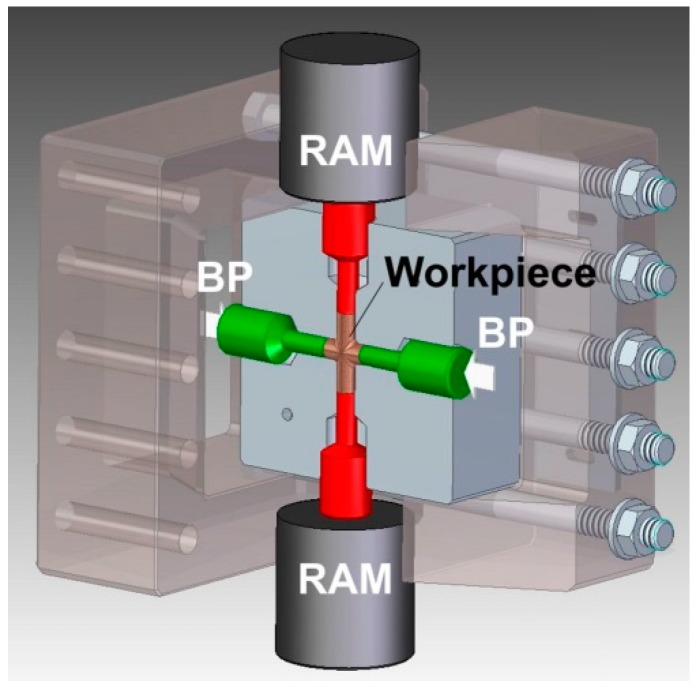
The die for cross-channel extrusion with back pressure (BP).

The tool assembly used for experimental trials of the CCE process is shown in Figure 2. It consists of two equal 1.2080 (AISI D3) steel blocks that both have a cross-shaped route with a circle shape in cross-section after their assembly. The diameter of channels is 8 mm and 50 mm length. The channels in the intersection area are connected by an arc having a radius of 2 mm. Four cylinder punches are used to press material and match the four channels at each side of the die.

As mentioned earlier, the CCE method is predisposed for the processing of hardly deformable materials. Therefore, to verify the results of FEM model and PTM technique, tests were carried out on Fe-22Al-5Cr-0.1Zr-0.01B (at. %) alloy—called as Fe-22Al-Cr in this work. The material in as-cast state was obtained by vacuum melting of pure elements followed by casting into ceramic molds. The ingots of 8 mm diameter and 40 mm length were homogenized at 1100 °C for 10 h.

It is well known that every pass of SPD techniques already have a substantial effect on both the properties and the microstructure of the material. For this reason, one and two passes of CCE were applied to achieve a fine grain size. A samples were extruded and deformed at the speed of 2 mm/s at room temperature, without and with BP = 10 kN, which corresponds to a stress of 200 MPa. To reduce friction on the metal-tool interface, a dry graphite lubricant spray was used.

The microstructure of material in its initial and deformed state was characterized by a light optical microscopy (LOM-Eclipse MA200, Nickon, Japan), FEI Quanta^TM^ 3D field emission gun scanning electron microscope (FEG-SEM, Hillsboro, OR, USA) coupled with X-ray energy dispersive spectroscopy (EDS-EDAX^TM^, AMETEK, Inc., Berwyn, PA, USA) and automatic electron backscatter diffraction system (EBSD). The analysis was performed on mechanically polished longitudinal sections of samples. The effect of the applied processing on the mechanical properties of the material was evaluated in microhardness measurements, using the Vickers indenter (HMV-G, Shimadzu, Kyoto, Japan) loaded with 0.2 kg.

## 3. Simulation of Cross-Channel Extrusion—Results and Discussion

### 3.1. FEM Simulation

The FEM simulation shows that during the CCE process, a misalignment of the mesh at 45° occurs in odd passes, whereas the mesh is restored and straightened in even passes. It has been observed, that a densification of mesh lines in the axis zone of the outlet channel points to an accumulation of high strains in this area. The observed arrangement of the mesh lines is very similar to that reported for other CCE-deformed materials [9,10].

**Figure 3:**
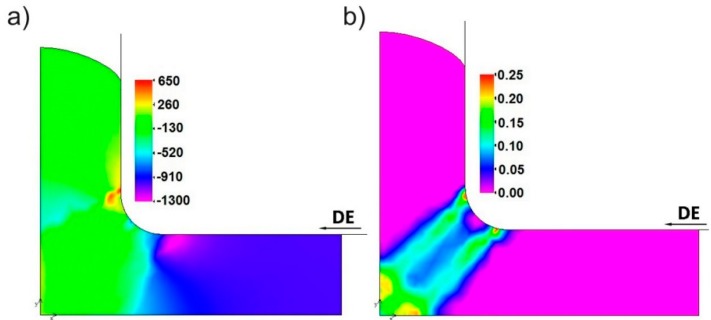
An example distributions of: (**a**) Stress [MPa]—corresponding to Figure 1, and strain rate [s^−1^] (**b**) during the 1st pass without back pressure (BP = 0 MPa).

A compressive stress (−900 MPa) is formed in the inlet channel (I-area in Figure 1b) of the die, while a passing of material to the outlet channel (III-area) leads to a sudden decrease in the compressive stress (−200 MPa) (Figure 3a). An adverse stress distribution was found in the outlet channel, near the C-point. A local presence of tensile stresses with a maximum value of 600 MPa, was observed in this region. This finding points to a possible presence of plastic flow instability in this area, which may lead to a material cracking.

Replacing the sharp internal corner with the ABC arc causes that the deformation is substantial only to the volume of material delimited by two twin planes A-D and C-F, oriented approximately 45° with respect to the axis of channels (II-area), as shown in Figure 3b. In these planes, the strain rate reaches a value of 0.1–0.2 s^−1^. A similar separation was observed by Perez and Luri [44], when a sharp internal corner of the channel die was replaced with an arc in the ECAP process. They observed that increasing the radius of 1.5 mm to 4 mm allows a more than 18% increase in the strain value to be obtained at a less than 9% increase of the processing force.

**Figure 4:**
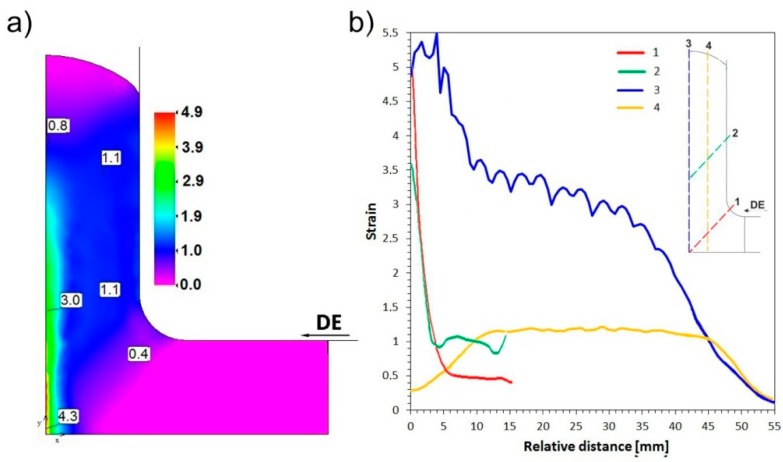
Equivalent plastic strain (*ε*): (**a**) Maps of distribution and (**b**) graph of its values at 1st pass, BP = 0 MPa.

As shown in Figure 4a, the distribution of equivalent plastic strain indicates that the extruded sample deforms substantially in the central-zone of the die, in the A-D and C-F planes (Figure 1b). A triaxial stress state dominates in the II-area, where the two channels intersect, leading to a strong increase of strain, even above *ε* = 5. Upon the movement of material into the output channel, this heavily deformed region is stretched to form a strip with a thickness of approx. 2.5 mm (in one quarter of the cross section) and a degree of deformation of 2 to 3 (IV-area). As seen at the beginning of line 4 (Figure 4b), the peak of strain corresponds to an area located directly under the active punch, associated with the triaxial compression (II-area), and the remaining volume of the material deforms by shear and is significantly easier shift in the output direction (III-area). This results in a relatively uniform strain of *ε* ≈ 1.1 in ¾ of its volume (lines 4). The wave visible on the graph most likely indicates discontinuous character of deformation. As in the ECAP, in each case a portion of the material deforms in shear and then displaced into the output channel [21,23,24]. This corresponds to slight fluctuations in deformation in the plane perpendicular to the extrusion direction.

Khan and Meredith [3] reported a formation of shear bands having several microns of width, oriented approximately 45° with respect to the pressure direction in Al 6061 that had been subjected to ECAP deformation. Zhao et al. [45] obtained similar results in a FEM simulation of the ECAP process, both in the case of a deformation behavior of the mesh grid and for the distribution of deformation. They indicate that the apparent heterogeneity of the deformation of the workpiece has been associated with friction on the bottom surface of the channel and its angle of channel refraction.

**Figure 5:**
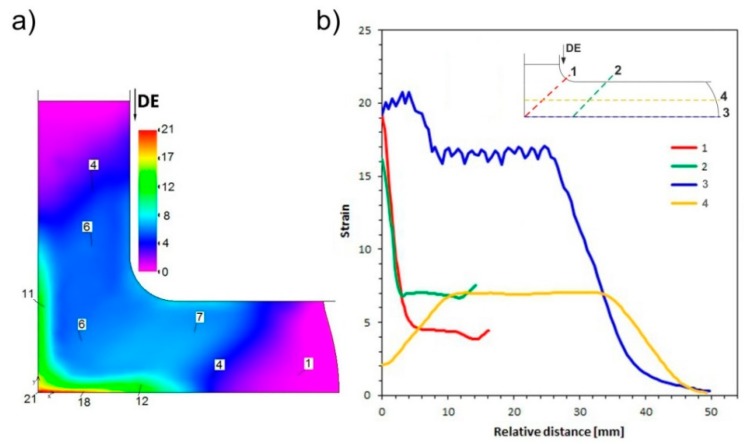
Equivalent plastic strain distribution (*ε*) at BP = 500 MPa: (**a**) First pass and (**b**) Sixth pass.

As mentioned above, this simulation was performed with a back pressure (BP) of 10% and 50% of the supposed extrusion pressure, which gives 100 MPa and 500 MPa, respectively. It should be noted that the introduction of back pressure does not substantially affect the nature of the stress distribution, but only causes its proportional increase corresponding to the variant without BP (Figure 5). The significant difference was observed for BP = 500 MPa, where behind the C-point in Figure 1b, the observed tensile stresses disappear or they change to compressive (Figure 5c). Therefore, BP = 100 MPa is an insufficient value, which should not allow a loss of flow continuity or the appearance of tensile stresses.

A strain rate distribution analysis showed a similar arrangement, as was the case for the BP absence—a substantial deformation occurs only in the A-D and C-F-diagonal planes. Regardless of the number of passes and the BP value, the instantaneous value does not exceed $\dot{\varepsilon }$ = 0.4 s^−1^, with a mean value of 0.15–0.2 s^−1^.

**Figure 6:**
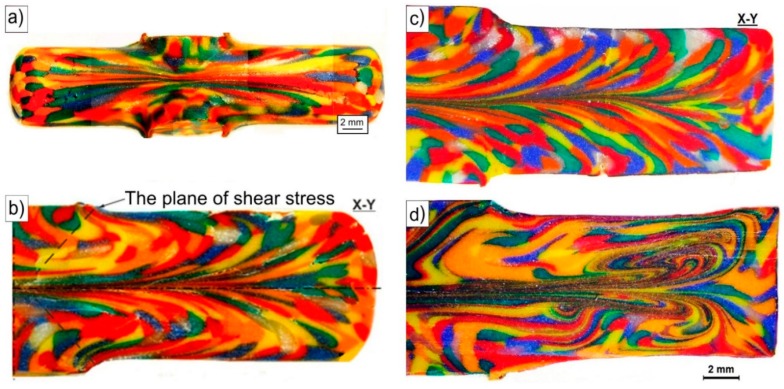
Modeling clay samples after: (**a**) One pass of CCE without BP and (**b**) cross-section of vertical-longitudinal samples and also (**c**) with back pressure—1 × CCE+BP, (**d**) 4 × CCE + BP.

An analysis of equivalent plastic strain distribution (Figure 6a) indicates its similar nature in the CCE extruded samples to samples deformed without back pressure. For both the BP = 100 and 500 MPa samples in the first pass, a strongly deformed zone (*ε* = 3.1–3.3) is formed around the sample axis (IV-area). The rest of the material undergoes a relatively homogeneous deformation of *ε* ≈ 1.1 (III-area), except for the tapered “locked” zone (V-area) located on the foreheads and central parts of the extruded sample (Figure 6b). In subsequent passes, an accumulation of strain leads to an increase in the *ε* value to 14–23 and 6–7 for the areas, as mentioned above. It is worth noting that the BP increasing to 500 MPa does not substantially affect the value of strain but does make it more homogeneous (Figure 6c). This is especially apparent in the IV-area, where the maximum and average value of strain were reduced (compared to *ε* = 21 and 16.6), with an unchanged strain value for the remaining volume of the material.

Hasani et al. [22] reported that when the BP-ECAP line-shaped flow of the material changes to a more rounded shape, the outer corner of channel matrix develops a dead-metal zone, accompanied by a twofold increase in the plastic deformation zone. It was due to the distribution of the strain rate, which reached a value of 0.35 s^−1^ for the material layers located directly at the internal corner of channel but reached only 0.2 s^−1^ in external corners. The introduction of BP (200 MPa) caused a decrease of rates and widening of the deformation zone.

Yoon et al. [24] conducted a FEM analysis of the ECAP process with and without BP. In the bottom zone of the sample, they observed the formation of a strongly deformed zone (*ε*~4), whereas in the remaining volume of material, the strain was lower (*ε*~1.1) and more homogeneous, which is in consensus with the results presented in this article. The implementation of BP reduced the gaps between the sample and the die in the outer corner of the channel, resulting in a strain increase at the sample’s bottom.

Djavanroodi and Ebrahimi [25] also suggested that the use of the BP provides a complete filling of the gap in the outer corner of the channel and reduces the dead zone fraction, thereby preventing the formation of cracks and improving the uniformity of stress distribution in the material.

The use of BP is not limited to the ECAP process, although it is the most common case. Zangiabadi and Kazeminezhad [26] developed a new deformation method that uses tube channel pressing. They noted that BP usage leads to a greater accumulation of strain in the material (approx. 1–1.2 per pass). The strain distribution in the longitudinal direction of the pipe has a high homogeneity in the presence of BP. Furthermore, the application of BP reduces the amount of waste material. Li et al. [46] used BP to achieve a full filling by the material during its extrusion through the die with a helical deformation zone in a mutative torsion extrusion channel method (MCTE).

### 3.2. Physical Simulation

Figure 6 shows images of modeling clay specimens processed by the CCE. After one pass, the clay grains undergo strong deformation. They are flattened and elongated in the extrusion direction (Figure 6a). Deviation of their axis to outer surfaces with the movement from the center towards the end face of the sample was observed. A small number of sample discontinuities is observed on the samples’ faces and on the outer surfaces near the C point in Figure 1b, which is compatible with FEM simulations.

The grains seen in the X-Y-section in the paraxial area underwent a strong deformation, resulting in the flattening or even blurring of their boundaries (Figure 6b). The effects of internal and the outer die wall friction are observed. This resulted in creation of a flat pattern along its axis, resembling the shape of the letter M lying on its side. In the perpendicular-section, the plane of strong deformation dissipates and a circular curvature appears as we move closer to the outer surface of the sample.

In summary, for CCE extrusion, the compressive and shearing stresses are responsible for the deformation of the material. The former affects the central part of the die (II-area) in the compression axis of the sample and causes severe flattening and elongation of the grains. The shear stresses are responsible for the deformation when the material moves towards the output channel and crosses the plane of their impact (A-D and F C-planes). They cause a disperse deformation of the remaining volume of the sample. The result is the deviation of the grains’ axis on the outside, which corresponds to the shear planes. It is suggested that the basic mechanism of shear deformation is the difference in the flow route or flow path induced by the geometric character of the ECAP die. Therefore, the structure of deformed material, depending on where it is located, corresponds to these two mechanisms or reflects the cross-matrix system, which is in good agreement with the results obtained from the FEM simulation.

The deformation character of the CCE process was investigated by Chou et al. [10,11]. To illustrate the deformation properties, they placed a square grid on the surface of a tin sample. After extrusion, it was deformed to form a symmetrical pattern whose lines converged at the central point of the sample and were inclined to the axis at an angle of 45°, as described above. This arrangement is similar to that formed in the ECAP by the action of shear stresses in the intersection zone of the channels. Assuming that the shape of the ECAP extruded sample by variant A corresponds to approximately ¼ of the one obtained from the CCE [1,3], we can compare the results.

Manna et al. [32] modeled the extrusion process based on plasticine and obtained a similar pattern. Its arcuate shape with anchored edges indicates a significant effect of friction in the deformation process. Wu and Backer [43] using a plexiglass mold, obtained a more uniform pattern, due to lower friction. By introducing lubricants, we can influence on the friction in the contact zone billet-die. Han et al. [41] extruded by ECAP the billet consisted of two kinds of grains color and a size of approximately 1 mm. The evolution of the flow patterns was governed by the geometric character of the die. The simple flow line field corresponds to the geometric aspect of shear deformation in the deformation zone of the billet during the experiments. Zhan et al. [33] modeled the process of forging a compressor blade using plasticine. They observed that as the process continued, the degree of non-uniformity of deformation of each blade increased.

Sofuoglu and Gedikli [39], comparing the results of the FEM simulation of the forward extrusion and its physical modeling based on plasticine, indicated their similarity, especially regarding emerging forces. The differences might be related to the adopted method of analysis of deformed grid patterns or to the inhomogeneity of the extrusion billet. Balasundar et al. [40] modeled the impact of the tool geometry on friction and constitutive relationship of materials *σ* = f(*ε*, $\dot{\varepsilon }$, T), which affect the material flow behavior, strain distribution and load requirement during the ECAP, and found their high compatibility with those of numerical simulations.

To simulate BP in the CCE process, the second set of pistons in the output channels was used. The resistance associated with their friction with the channel matrix walls reflects this pressure. The macroscopic observations of the deformed samples and of patterns formed on their outer surface did not differ substantially from those described previously. The shape of the sample reflects the exact shape of the channel die without any cracking or chipping. In the internal structure (Figure 6c), only the grains in the paraxial zone (IV-area) seemed to be more flattened and elongated with an expansion of this zone to the outer surface (area III). However, in the immediate vicinity, there are equiaxed grains. The curvature of their shape in the direction opposite to the flow is probably associated with the die wall friction.

With an increase in the number of passes, the microstructure of the CCE extruded clay underwent further development and became more complex. After four passes with BP (Figure 6d), a band structure was formed in the paraxial zone (IV-area). It consisted of highly flattened and very elongated grains, which boundaries practically blurred. Repeated of extrusion led to the curvature, looping and expansion of these bands and thus to increase of deformation homogeneity.

Manna et al. [32] modeled an extrusion process based on plasticine with as many as 15 passes and observed a significant mixing of ingredients of plasticine with a drastic reduction in the size of each section.

### 3.3. The Cross-Channel Extrusion of Fe-22Al-5Cr Alloy

**Figure 7:**
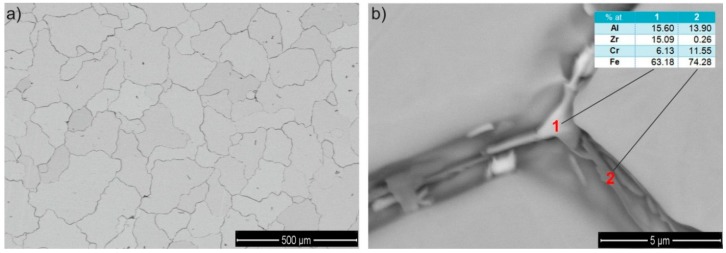
The initial microstructure of as-cast and homogenized Fe-22Al-5Cr alloy (**a**) and the chemical composition of precipitates at grain boundaries (**b**).

The microstructure of the starting material is shown in Figure 7. The structure is characterized by the presence of medium sized equiaxed grains. The average grain size (*d_eq_*) equals about 193 µm. X-ray phase analysis shows that the alloy has a single-phase structure based on a disordered solid solution of aluminum in α-iron with A2 lattice. However, a detailed BSE analysis proved that there are chromium or zirconium-rich precipitates on the grain boundaries GB (Figure 7b), which may be described as the (Fe,Al)_2_Zr Laves phase [47]. Using a static tension test, it was found that the value of yield strength is 480 MPa, tensile strength 470 MPa, and a total elongation 1.5%.

**Figure 8:**
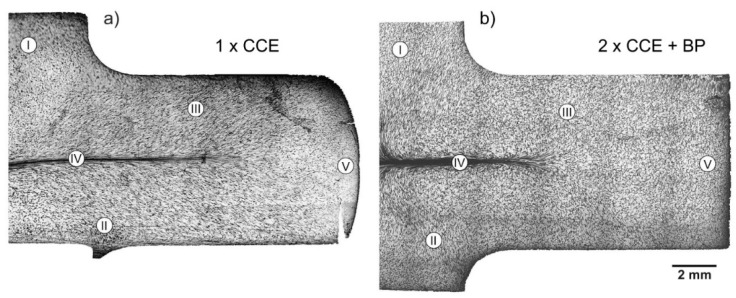
Cross-section of deformed alloy after: (**a**) One pass without BP and (**b**) two passes with BP = 10 kN of CCE. I–V-areas as Figure 1b.

**Figure 9:**
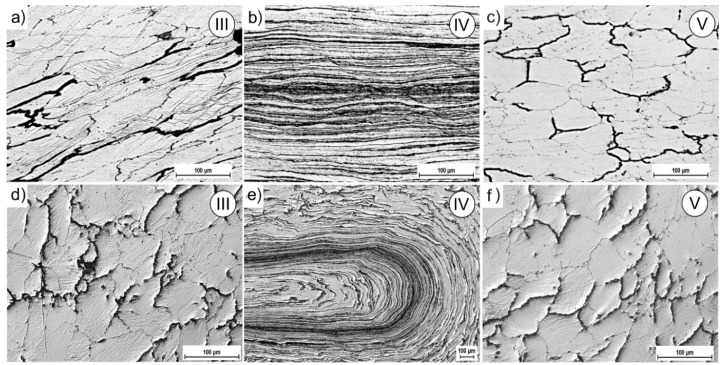
Microstructure of Fe-22Al-5Cr alloy from Figure 8: (**a**–**c**) One pass and (**d**–**f**) two passes of CCE.

The macro-OIM-s after CCE process are presented in Figure 8, which shows parallel plane to the extrusion direction. The macrostructure of the samples maps the stress state caused by the cross channel system. Referring to earlier analyses, we can distinguish three types of structures (Figure 9).

The first, associated with shear stresses, is located on both sides of longitudinal axis of the sample and corresponds to the III-area. The structure of the material is strongly deformed with very many slip bands (Figure 9a). The grains (*d_eq_* ≈ 60 µm) are elongated and their direction of deflection towards the external surfaces is associated with the passage through the shear plane in II-area (see Figure 1b). The second, with a very strong deformation, creates a characteristic band along the *X*-axis of the extruded sample (Figure 9b). This corresponds to the IV-area, where we observed a band structure. The grains are strongly elongated with a blurring of the boundaries. The width of these bands, and therefore the grains is 1–5 µm. The third, associated with blocked stresses, was observed in tapered zones under the piston (I-area) or on foreheads of the sample (V-area). The grains are equi-axial and their size decreases almost twice from the initial state in this zone (Figure 9c).

In the next passage, the deformation increases and undergo propagates, which leads to a more uniform distribution. As a result of re-passing through the shear plane, the grains return to equiaxed, especially in the III-area (Figure 9d), which is consistent with previous reports [10,48,49]. The structure becomes slightly more fine-grained. The bands width in the IV-area is reduced, but as a result of the sample rotated by 90° between the first and second pass, we can observe their characteristic looping (Figure 9e), as in the case of clay modeling (Figure 6d).

After one pass of CCE, there were no cracks observed in the material volume except for the area (V) on the surface of the extruded sample. The BP’s introduction practically eliminated cracks and improved the shape of the sample.

**Figure 10:**
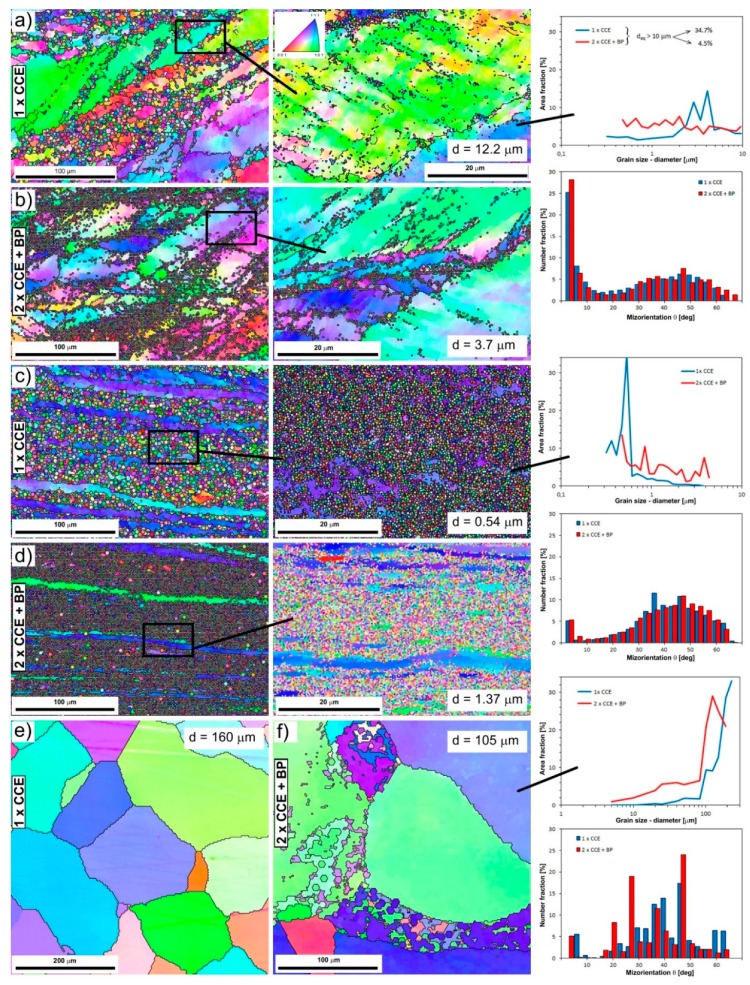
Electron backscatter diffraction system (EBSD) misorientation maps (OIM) obtained from samples subjected to CCE process with different areas (Figure 1b): (**a**,**b**) III-area of cutting, (**c**,**d**) IV-area of triaxial compression (**e**,**f**) V-area of blocked stresses, at one pass of CCE without BP and two passes with BP. Black lines of the colored areas outline correspond to the high-angular nature of grain boundaries (HAGB).

Typical EBSD maps of boundaries misorientation and grain size distribution of the as-CCE deformed microstructure are shown in Figure 10. As mentioned earlier, the structure of material deformed once by CCE in the area of the influence of shear stresses (III-area) consists of racked and elongated primary grains (Figure 10a). Because of a non-continuous process, their volume is traversed by numerous slip bands along where new grains are recrystallized with a size less than a few micrometers. Depending on the conditions, even whole grains may undergo refining. However, there is still a significant, almost 35% share of grains above 10 µm. Therefore, we are dealing with a typical bimodal distribution, characteristic of the processes associated with dynamic recrystallization of the material. Share of grains with a low disorientation’s angle (low angle grains boundaries LAGB: θ < 15°) in this process is about 40%, the highest share constitute of grains for which the angle is about 40–50° (high angle grains boundaries HAGB: θ > 15°). Increase of deformation, as a result of repeated passes, causes a significant growth in the number of grains 0.3–2 µm, which takes place mainly at the expense of the remaining large primary grains. The actual size of the grains may be even smaller, but it would require the use of TEM testing [50].

The three-axis compressing occurring in the central part of the sample is responsible for the formation of a band structure along the *X*-axis (Figure 10c). Grain-bands about even a few hundred microns in length and just over a dozen microns wide are visible inside it. The changing colors inside them evidence of deformation of their lattice structure, so with a high value of the misorientation angle. However, the new, fully recrystallized, equi-axial grains with a size of 400–500 nm dominate in the structure. As a result, we are dealing with single mode the high-angular distribution of boundaries. With the next passage, as in the case of III-area, there is a general increase in the amount of fine grains in a wide range of size.

However, the material located in conical zones under the piston (I-area) or on the forehead of the extruded sample (V-area) is subject to much less impact, and thus to less deformation. This happens because it is not affected by cutting stresses or triaxial compression. Moreover, but on a much smaller scale, nucleation of new grains occurs and the size of the primary grains is reduced (Figure 10f). Thus, the present microstructural observation clearly displayed that CCE processing is able to refine the grain size of Fe-22A-5Cr alloy to UFG level.

At the same time, it seems that the introduction of BP = 200 MPa, which corresponded to 20% of the main pressing pressure, for such a hardly deformable material at RT as the Fe-22Al-5Cr alloy, it does not have a significant effect on the above-described structural changes as a result of CCE process. Undoubtedly, the application of BP leads to the suppression of a sample’s damage and closure of defects, whereas its absence leads to the development of defects due to the imposed severe plastic deformation, as also Lapovok pointed out [21]. However, it seems that this impact is largely associated with material parameters [22], and therefore, it is necessary to carry out a systematic examination of the role of a BP during grain refinement.

In the described case, rebuilding of the deformed material structure occurs due to the fragmentation of its grain under the influence of bands and microshear bands (MSBs) at low temperatures below 0.5 Tm. The appearance of MSB in deformed material leads to internal structural instability, as described by Sitdikov et al. [51]. With the increasing of deformation, new ultrafine grains appear first in the MSB or at their intersections, and only then inside the grain. This is accompanied by the rotation of the crystal lattice and increase of the misorientation angle, up to formation of HAGB. Thus, the multiple deformation associated with the change in the strain path causes the material to develop a spatial network of strain-induced boundaries containing both LAGB (subgrains) and HABS [52]. A prerequisite for full refinement grain (UFG) is to create a state to achieve the maximum density of MSBs and their possible frequent intersection, which is possible to achieve only in SPD processes. Therefore, the CCE process involving multi-directional shear combined with triaxial compression speeds up the process of fragmentation of the material structure. The partial primary recrystallization it is probably caused by the inhomogeneous distributions of deformation strain, especially in III-area. Even for the relatively equiaxed grain structure after 2 passes, microstructures are also inhomogeneous. However, the resulting small grains are recrystallized grains formed during the CCE process, because they are free of dislocations, as in [53].

With further deformation (increasing the number of passes) most of the LAGB evolve into HAGB leading to homogeneity and an ultrafine-grain material, as results of continuous dynamic recrystallization [22,54,55].

**Figure 11:**
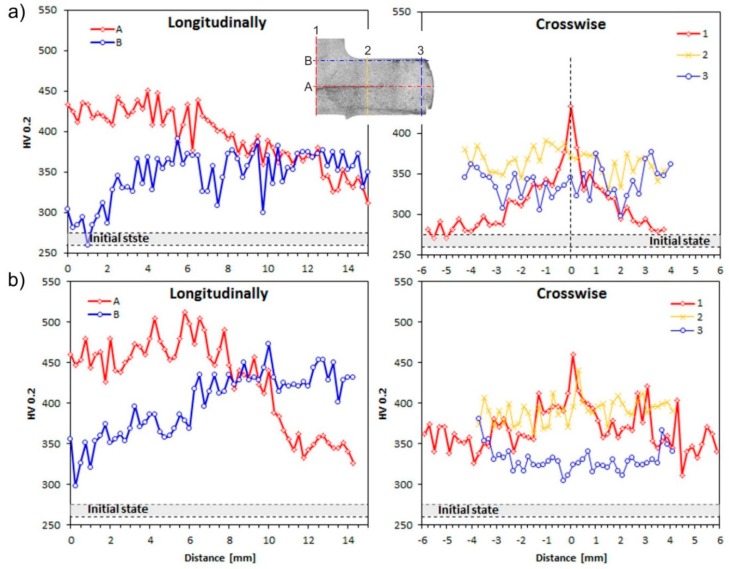
Microhardness of deformed alloy after: (**a**) One and (**b**) two passes of CCE.

The microhardness results along a longitudinal section of the samples show significant differences depending upon the area and conditions of deformation. The microhardness has hardly changed in I and V-area and remains at the level of 285 HV0.2 after one pass of CCE (Figure 11a,b), which is related with the lack of severe deformation mechanisms in this field. While, the transition through the plane of shear stress causes microhardness increasing to 370 HV0.2 in III-area and achieve a maximum 450 HV0.2 in IV-area. Interestingly, while the structure of the material in I and V areas has not undergone significant changes, the hardness in V-area increases after two passes. The microhardness of the tested alloy increases by an average of 10% (Figure 11b). This behavior is typical for metallic materials, especially on iron, subjected to deformation by SPD techniques [50,54,55,56,57]. Generally, the level of hardness is increased with the increase in the number of passes and the complexity of the stresses.

These observations remain in full agreement with the previously described results for the extrusion of modeling clay.

## 4. Conclusions

To determine the behavior of the Fe-22Al-5Cr alloy during CCE process conducted with or without back pressure, the appropriate thermodynamic calculations, FEM/physical simulations and experimental analysis has been developed.

From the FEM analysis, it is observed that a movement of the material in opposite directions in the cross-channel leads to the formation of complex stress systems. A blocking of the material and its triaxial compression occur near the central point of the die. As a consequence, a strongly deformed paraxial zone (*ε* ≈ 5) is formed along a channel axis perpendicular to the extrusion direction. It covers approx. 10–15% of the volume of the material. As the material is pushed to the outlet channel, the shear stress grows rapidly, which provides a relatively homogeneous deformation of approximately 1.1 in the remaining volume. The theoretical possibility of infinite accumulation of the effects of deformation in subsequent passes (*ε* ≈ 20/6.9 after 6 passes) and cyclic changes in the strain paths will undoubtedly lead to a strong fragmentation of the initial structure of the material and an improvement of its mechanical properties. The introduction of BP gives a positive effect on extrusion process by reducing of tensile stress and maintaining material integrity. This prevents the material from cracking and enables the processing of materials with a limited ductility. The BP has essentially no effect on the strain rate and strain values, excluding their more homogeneous distribution.

Physical modeling investigation reveals that the structure of deformed material corresponds to two mechanisms (the compressive and shearing stresses) or reflects the cross-matrix system. The first of these leads to the formation of a band structure, while the second cause shear and looping of the structure, progressing with numbers of passes.

The CEE process carried out on Fe-22Al-5Cr (at. %) alloy confirmed the results obtained by physical modeling, which are also in good agreement with the results obtained from the FEM simulation. The effect of the CCE process is the fragmentation of the original material structure by dividing the primary grains, due to the generation under the influence of shear stresses and the microshear bands and triaxial compression of the material in its central part. The complexity of the stress state leads to the rapid growth of MSB, grain defragmentation and the nucleation of new dynamically recrystallized grains. Their size ranges from 200–400 nm.

The BP improves the homogeneity of deformation, while not substantially affecting its other structure transformations, in terms of the used parameters. It leads to the suppression of sample damage and closure of defects.

The results also indicate a need for further works related to the optimization of the shape and dimensions of each die’s channel.
